# 6-Bromo­pyridine-2-carboxamide

**DOI:** 10.1107/S1600536809047114

**Published:** 2009-11-21

**Authors:** Feng Xue, Shen-gui Ju

**Affiliations:** a College of Chemistry and Chemical Engineering, Nanjing University of Technology, Xinmofan Road No. 5 Nanjing, Nanjing 210009, People’s Republic of China

## Abstract

In the the title compound, C_6_H_5_BrN_2_O, an intra­molecular N—H⋯N hydrogen bond generates an *S*(5) ring. In the crystal structure, inter­molecular bifurcated N—H⋯(O,O) hydrogen bonds link the mol­ecules, leading to sheets propagating in (100).

## Related literature

For medicinal background to inhibitors of the crysteine protease cathepsin K, see: Altmann & Aichholz (2007 ▶).
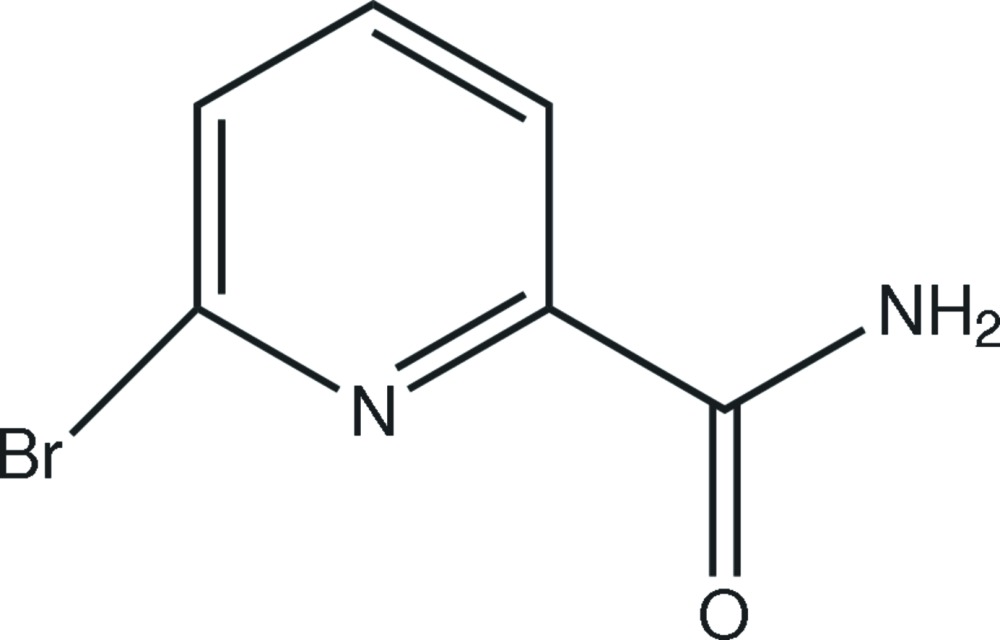


## Experimental

### 

#### Crystal data

C_6_H_5_BrN_2_O
*M* *_r_* = 201.03
Monoclinic, 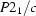

*a* = 13.034 (3) Å
*b* = 6.4050 (13) Å
*c* = 8.5540 (17) Å
β = 94.85 (3)°
*V* = 711.6 (2) Å^3^
*Z* = 4
Mo *K*α radiation
μ = 5.70 mm^−1^
*T* = 293 K
0.20 × 0.10 × 0.10 mm

#### Data collection

Enraf–Nonius CAD-4 diffractometer
Absorption correction: ψ scan (North *et al.*, 1968 ▶) *T*_min_ = 0.395, *T*_max_ = 0.599
1354 measured reflections
1296 independent reflections
756 reflections with *I* > 2σ(*I*)
*R*_int_ = 0.063
3 standard reflections every 200 reflections intensity decay: 1%

#### Refinement

*R*[*F* ^2^ > 2σ(*F* ^2^)] = 0.063
*wR*(*F* ^2^) = 0.172
*S* = 1.01
1296 reflections
91 parameters
H-atom parameters constrained
Δρ_max_ = 0.45 e Å^−3^
Δρ_min_ = −0.57 e Å^−3^

### 

Data collection: *CAD-4 EXPRESS* (Enraf–Nonius, 1994 ▶); cell refinement: *CAD-4 EXPRESS*; data reduction: *XCAD4* (Harms & Wocadlo, 1995 ▶); program(s) used to solve structure: *SHELXS97* (Sheldrick, 2008 ▶); program(s) used to refine structure: *SHELXL97* (Sheldrick, 2008 ▶); molecular graphics: *SHELXTL* (Sheldrick, 2008 ▶); software used to prepare material for publication: *PLATON* (Spek, 2009 ▶).

## Supplementary Material

Crystal structure: contains datablocks global, I. DOI: 10.1107/S1600536809047114/hb5211sup1.cif

Structure factors: contains datablocks I. DOI: 10.1107/S1600536809047114/hb5211Isup2.hkl

Additional supplementary materials:  crystallographic information; 3D view; checkCIF report

## Figures and Tables

**Table 1:** Hydrogen-bond geometry (Å, °)

*D*—H⋯*A*	*D*—H	H⋯*A*	*D*⋯*A*	*D*—H⋯*A*
N2—H2*B*⋯N1	0.86	2.41	2.730 (10)	102
N2—H2*A*⋯O^i^	0.86	1.99	2.849 (9)	176
N2—H2*B*⋯O^ii^	0.86	2.22	3.002 (9)	151

Symmetry codes: (i) 
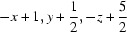
; (ii) 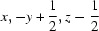.

**Fig. 1.**
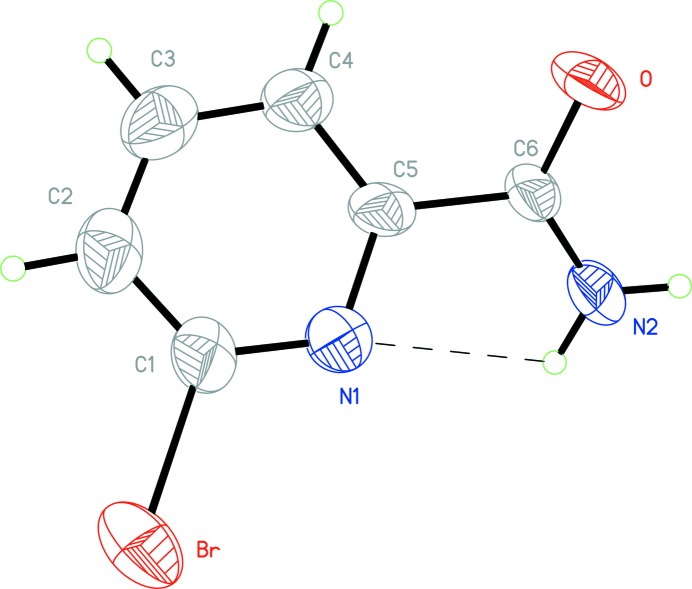
The molecular structure of (I) showing 50% displacement ellipsoids.

**Fig. 2.**
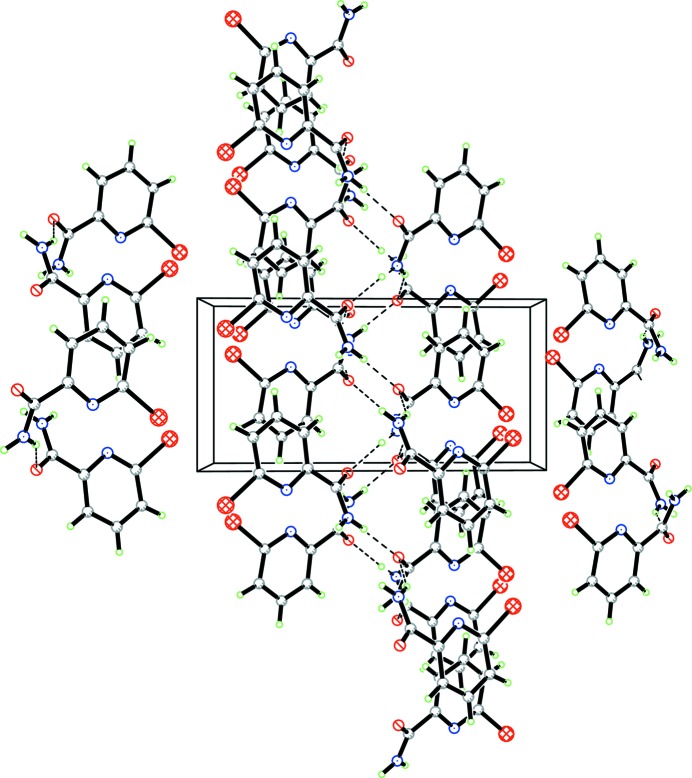
A partial packing diagram of (I). Hydrogen bonds are shown as dashed lines.

## supplementary crystallographic information

### Experimental

A mixture of 30 g of 6-bromopyridine-2-carboxylic acid (0.1485 mol) in 300 ml of thionyl chloide was refluxed for twenty hours. Excess thionyl chloride was removed in vacuo. The residue was added as a slurry in dioxane or benene to 75 ml cold, stirred concentrated ammonium hydroxide. The mixture was stored overnight and the filtered to give 25 g of the title compound (yield 83.4%, m.p. 417 K). Colourless blocks of (I) were obtained by the slow evaporation of an ethyl acetate solution.

### Refinement

H atoms were positioned geometrically, with C-H = 0.93–0.97Å, and refined as riding with U_iso_(H) = 1.2U_eq_(C) or 1.5U_eq_(methyl C).

### Crystal data

**Table:**

C_6_H_5_BrN_2_O	*F*(000) = 392
*M**_r_* = 201.03	*D*_x_ = 1.877 Mg m^−^^3^
Monoclinic, *P*2_1_/*c*	Melting point: 417 K
Hall symbol: -P 2ybc	Mo *K*α radiation, λ = 0.71073 Å
*a* = 13.034 (3) Å	Cell parameters from 25 reflections
*b* = 6.4050 (13) Å	θ = 9–12°
*c* = 8.5540 (17) Å	µ = 5.70 mm^−^^1^
β = 94.85 (3)°	*T* = 293 K
*V* = 711.6 (2) Å^3^	Block, colourless
*Z* = 4	0.20 × 0.10 × 0.10 mm

### Data collection

**Table:**

Enraf–Nonius CAD-4 diffractometer	756 reflections with *I* > 2σ(*I*)
Radiation source: fine-focus sealed tube	*R*_int_ = 0.063
graphite	θ_max_ = 25.3°, θ_min_ = 1.6°
ω/2θ scans	*h* = −15→0
Absorption correction: ψ scan (North *et al.*, 1968)	*k* = 0→7
*T*_min_ = 0.395, *T*_max_ = 0.599	*l* = −10→10
1354 measured reflections	3 standard reflections every 200 reflections
1296 independent reflections	intensity decay: 1%

### Refinement

**Table:**

Refinement on *F*^2^	Primary atom site location: structure-invariant direct methods
Least-squares matrix: full	Secondary atom site location: difference Fourier map
*R*[*F*^2^ > 2σ(*F*^2^)] = 0.063	Hydrogen site location: inferred from neighbouring sites
*wR*(*F*^2^) = 0.172	H-atom parameters constrained
*S* = 1.01	*w* = 1/[σ^2^(*F*_o_^2^) + (0.095*P*)^2^] where *P* = (*F*_o_^2^ + 2*F*_c_^2^)/3
1296 reflections	(Δ/σ)_max_ < 0.001
91 parameters	Δρ_max_ = 0.45 e Å^−^^3^
0 restraints	Δρ_min_ = −0.57 e Å^−^^3^

### Special details

**Table:**

Geometry. All esds (except the esd in the dihedral angle between two l.s. planes) are estimated using the full covariance matrix. The cell esds are taken into account individually in the estimation of esds in distances, angles and torsion angles; correlations between esds in cell parameters are only used when they are defined by crystal symmetry. An approximate (isotropic) treatment of cell esds is used for estimating esds involving l.s. planes.
Refinement. Refinement of F^2^ against ALL reflections. The weighted R-factor wR and goodness of fit S are based on F^2^, conventional R-factors R are based on F, with F set to zero for negative F^2^. The threshold expression of F^2^ > 2sigma(F^2^) is used only for calculating R-factors(gt) etc. and is not relevant to the choice of reflections for refinement. R-factors based on F^2^ are statistically about twice as large as those based on F, and R- factors based on ALL data will be even larger.

### Fractional atomic coordinates and isotropic or equivalent isotropic displacement parameters (Å^2^)

**Table:**

	*x*	*y*	*z*	*U*_iso_*/*U*_eq_	
Br	0.91125 (7)	0.18241 (18)	0.81361 (11)	0.0756 (5)	
O	0.5752 (4)	0.0268 (9)	1.2691 (6)	0.0584 (15)	
N1	0.7461 (5)	0.1078 (10)	0.9836 (6)	0.0440 (16)	
N2	0.5728 (6)	0.2794 (11)	1.0883 (8)	0.061 (2)	
H2A	0.5265	0.3491	1.1317	0.073*	
H2B	0.5970	0.3260	1.0045	0.073*	
C1	0.8216 (6)	0.0086 (14)	0.9207 (8)	0.049 (2)	
C2	0.8389 (7)	−0.2007 (15)	0.9279 (9)	0.057 (2)	
H2C	0.8929	−0.2615	0.8798	0.068*	
C3	0.7743 (7)	−0.3160 (14)	1.0077 (10)	0.059 (2)	
H3A	0.7813	−0.4605	1.0110	0.071*	
C4	0.6990 (7)	−0.2223 (12)	1.0837 (9)	0.052 (2)	
H4A	0.6566	−0.2999	1.1439	0.062*	
C5	0.6875 (6)	−0.0119 (11)	1.0690 (7)	0.0408 (18)	
C6	0.6063 (6)	0.1033 (13)	1.1493 (8)	0.0417 (18)	

### Atomic displacement parameters (Å^2^)

**Table:**

	*U*^11^	*U*^22^	*U*^33^	*U*^12^	*U*^13^	*U*^23^
Br	0.0750 (7)	0.0972 (9)	0.0596 (7)	−0.0039 (6)	0.0341 (5)	0.0056 (6)
O	0.084 (4)	0.056 (3)	0.039 (3)	−0.012 (3)	0.031 (3)	0.006 (3)
N1	0.063 (4)	0.046 (4)	0.023 (3)	0.000 (3)	0.007 (3)	−0.002 (3)
N2	0.088 (5)	0.057 (4)	0.044 (4)	0.014 (4)	0.039 (4)	0.011 (4)
C1	0.065 (5)	0.058 (6)	0.025 (4)	0.012 (5)	0.010 (3)	0.001 (4)
C2	0.068 (5)	0.063 (6)	0.038 (5)	0.015 (5)	0.008 (4)	−0.008 (4)
C3	0.089 (6)	0.045 (5)	0.044 (5)	0.006 (5)	0.005 (5)	−0.004 (4)
C4	0.077 (5)	0.042 (5)	0.037 (4)	−0.002 (5)	0.012 (4)	0.000 (4)
C5	0.061 (5)	0.038 (4)	0.024 (4)	−0.005 (4)	0.009 (3)	0.003 (3)
C6	0.057 (5)	0.038 (4)	0.032 (4)	−0.007 (4)	0.020 (3)	−0.006 (4)

### Geometric parameters (Å, °)

**Table:**

Br—C1	1.905 (8)	C2—C3	1.349 (12)
O—C6	1.235 (8)	C2—H2C	0.9300
N1—C1	1.322 (9)	C3—C4	1.362 (12)
N1—C5	1.342 (9)	C3—H3A	0.9300
N2—C6	1.303 (10)	C4—C5	1.361 (10)
N2—H2A	0.8600	C4—H4A	0.9300
N2—H2B	0.8600	C5—C6	1.503 (10)
C1—C2	1.360 (11)		
			
C1—N1—C5	115.1 (6)	C2—C3—H3A	119.8
C6—N2—H2A	120.0	C4—C3—H3A	119.8
C6—N2—H2B	120.0	C5—C4—C3	118.1 (8)
H2A—N2—H2B	120.0	C5—C4—H4A	121.0
N1—C1—C2	125.6 (8)	C3—C4—H4A	121.0
N1—C1—Br	115.0 (6)	N1—C5—C4	123.6 (7)
C2—C1—Br	119.4 (6)	N1—C5—C6	115.1 (6)
C3—C2—C1	117.0 (8)	C4—C5—C6	121.4 (7)
C3—C2—H2C	121.5	O—C6—N2	123.6 (7)
C1—C2—H2C	121.5	O—C6—C5	118.5 (7)
C2—C3—C4	120.4 (8)	N2—C6—C5	117.8 (6)
			
C5—N1—C1—C2	4.5 (11)	C1—N1—C5—C6	176.1 (6)
C5—N1—C1—Br	−175.2 (5)	C3—C4—C5—N1	0.2 (12)
N1—C1—C2—C3	−0.9 (13)	C3—C4—C5—C6	−179.9 (7)
Br—C1—C2—C3	178.7 (6)	N1—C5—C6—O	−154.5 (7)
C1—C2—C3—C4	−3.3 (13)	C4—C5—C6—O	25.5 (11)
C2—C3—C4—C5	3.6 (13)	N1—C5—C6—N2	25.3 (10)
C1—N1—C5—C4	−4.0 (10)	C4—C5—C6—N2	−154.6 (8)

### Hydrogen-bond geometry (Å, °)

**Table:**

*D*—H···*A*	*D*—H	H···*A*	*D*···*A*	*D*—H···*A*
N2—H2B···N1	0.86	2.41	2.730 (10)	102
N2—H2A···O^i^	0.86	1.99	2.849 (9)	176
N2—H2B···O^ii^	0.86	2.22	3.002 (9)	151

Symmetry codes: (i) −*x*+1, *y*+1/2, −*z*+5/2; (ii) *x*, −*y*+1/2, *z*−1/2.
